# T Helper 17/Regulatory T Cell Balance and Experimental Models of Peritoneal Dialysis-Induced Damage

**DOI:** 10.1155/2015/416480

**Published:** 2015-05-03

**Authors:** Georgios Liappas, Guadalupe Tirma Gónzalez-Mateo, Pedro Majano, José Antonio Sánchez- Tomero, Marta Ruiz-Ortega, Raquel Rodrigues Díez, Pilar Martín, Raquel Sanchez-Díaz, Rafael Selgas, Manuel López-Cabrera, Abelardo Aguilera Peralta

**Affiliations:** ^1^Department of Immunology, Centro de Biología Molecular Severo Ochoa (CBM), Consejo Superior de Investigaciones Científicas (CSIC), 28049 Madrid, Spain; ^2^Department of Nephrology, Instituto de Investigación del Hospital Universitario La Paz (IdiPAZ), 28046 Madrid, Spain; ^3^Department of Molecular Biology, Instituto de La Princesa (IP), 28006 Madrid, Spain; ^4^Nephrology Service, Hospital Universitario de La Princesa (IP), 28006 Madrid, Spain; ^5^Laboratory of Cellular Biology and Rare Diseases, Fundación Jiménez Díaz, Universidad Autónoma de Madrid (UAM), 28040 Madrid, Spain; ^6^Department of Molecular and Vascular Inflammation, Centro Nacional de Investigaciones Cardiovasculares (CNIC), 28029 Madrid, Spain

## Abstract

Fibrosis is a general complication in many diseases. It is the main complication during peritoneal dialysis (PD) treatment, a therapy for renal failure disease. Local inflammation and mesothelial to mesenchymal transition (MMT) are well known key phenomena in peritoneal damage during PD. New data suggest that, in the peritoneal cavity, inflammatory changes may be regulated at least in part by a delicate balance between T helper 17 and regulatory T cells. This paper briefly reviews the implication of the Th17/Treg-axis in fibrotic diseases. Moreover, it compares current evidences described in PD animal experimental models, indicating a loss of Th17/Treg balance (Th17 predominance) leading to peritoneal damage during PD. In addition, considering the new clinical and animal experimental data, new therapeutic strategies to reduce the Th17 response and increase the regulatory T response are proposed. Thus, future goals should be to develop new clinical biomarkers to reverse this immune misbalance and reduce peritoneal fibrosis in PD.

## 1. Introduction

An effective inflammatory response is essential not only for the resolution of infections but also for wound healing after injury. The repair is mediated by the collaboration of various mechanisms launched by an acute inflammatory reaction. This process implicates the release of chemokines and cytokines and the migration of various cells of the immune system. If a sustained inflammatory reaction that is not resolved properly becomes chronic, it could lead to fibrosis due to the accumulation of extracellular matrix (ECM) components [1].

**Figure 1:**
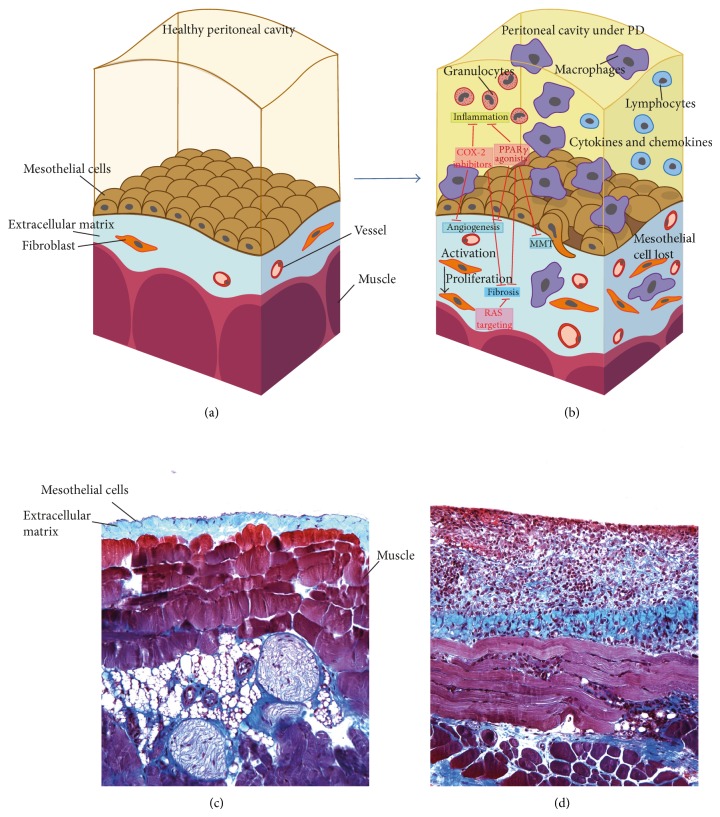
PM schemas and biopsies representing the normal peritoneal structure and its changes during PD. Possible therapeutic approaches. (a) and (b) are adapted with permission from Aguilera et al. (2013).* Available from* http://www.intechopen.com/books/the-latest-in-peritoneal-dialysis/the-mesothelial-to-mesenchymal-transition-a-pathogenic-and-therapeutic-key-for-peritoneal-membrane-f [80]. (a) Realistic representation of a healthy peritoneal cavity. A preservation of the mesothelial layer is clearly seen, only a few fibroblasts and vessels are visible, and only a small layer of extracellular matrix lies in the compact zone of the muscle layer. (b) After exposure to PD liquids, the structure of the PM starts to change dramatically with the appearance of more fibroblasts, macrophages, and inflammatory cytokines and finally with deposition of more extracellular matrix (ECM) cells. Inflammation and fibrosis will be the result of these changes. Some drugs like COX-2 inhibitors, PPRA*γ* agonists, or RAS targeting are possible therapeutic strategies to protect from PD complications such as inflammation, angiogenesis, fibrosis, and/or MMT. (c) A peritoneal biopsy of a mouse peritoneal membrane that was treated with physiological saline is shown as control. The PM is well preserved and fibrotic response is absent. (d) Peritoneal biopsy of a mouse PM that was exposed to PDF for 40 days. A significant fibrotic response can be observed with a larger ECM and many inflammatory cells.

Peritoneal dialysis (PD) is a form of renal replacement therapy alternative to haemodialysis that is widely used around the world for patients suffering from renal failure disease [2, 3]. The process uses the peritoneum as a semipermeable membrane across which PD fluids (PDFs) and dissolved substances (electrolytes, urea, glucose, and other small molecules) are exchanged from the blood [4]. The peritoneal membrane (PM) acts as a protective barrier against injury and pathogens, where humoural and cellular responses are generated. The treatment consists of the instillation and periodical renovation of a hyperosmotic PDF in the peritoneal cavity through a permanent installed catheter. However, the mechanical damage due to PDF instillation and the exposure of peritoneal cells to glucose degradation products (GDPs) and advanced glycation end products (AGEs) (due to the nonphysiological nature of this PDF) generates inflammation. Along these lines, it has been demonstrated* in vitro* that GDPs and AGEs stimulate NF*κ*B-mediated transcription and the secretion of cytokines and chemokines by human peritoneal mesothelial cells [5]. In addition, the presence of the catheter [6] and peritonitis episodes during treatment [7] appears to be responsible for various alterations of the PM structure and functionality. The final consequence is the generation of vascular alterations [8] and peritoneal fibrosis [3, 9], leading to an ultrafiltration failure [10, 11] that impedes the dialysis process (Figure 1). Although in recent decades great effort has been made to improve catheter design [6] and the biocompatible solutions used [12–14], complications are still common [15, 16]. It has also been demonstrated that inflammation is present in PD patients. Increased serum concentrations of IL-6, TNF-*α*, VEGF, and C-reactive proteins have been reported in patients, suggesting that PD leads to increased systemic inflammation [17]. Moreover, there is evidence in animal models of chronic peritoneal exposure to PDF showing that this procedure induces PM inflammation and fibrosis [18]. The use of an anti-inflammatory drug (a Cox-2 inhibitor) reduced this peritoneal inflammatory response and consequently the fibrosis. This result confirms the role of inflammation in peritoneal fibrosis [19].

Inflammation is driven by various cell populations including macrophages, neutrophils, and lymphocyte subsets. The differentiation of T cells is crucial for immune and inflammatory responses and its regulation may be a therapeutic target to control peritoneal damage. It has been found that there are different rates between CD4^+^ and CD8^+^ cells in the peritoneum during PD with respect to healthy individuals [20]. It has been postulated that the presence of AGEs is responsible for an increase in the population of CD8^+^ (T cytotoxic) lymphocytes [21]. Regarding CD4^+^ (T helper) subsets, in general terms Th1 cells produce high levels of IFN-*γ*, while Th2 cells secrete predominantly IL-4 [22]. There is some controversy about the pattern of response that is generated in patients undergoing treatment with PDF. It is described that there is a deviation toward Th2 pattern of PD in stable patients [23]. However, during episodes of acute peritonitis, a Th1 immune response is developed [24, 25]. In addition to these two classical T helper cell subsets (Th1 and Th2), a third and fourth subpopulation, designated regulatory T (Treg) and T helper 17 (Th17) cells, have emerged as independent differentiation pathways [26, 27]. While the predominance of Th17 cells induces the secretion of a large number of proinflammatory cytokines, Treg cells restrict inflammatory responses and are associated with immune-tolerance [28]. Very little is known about the involvement of these subpopulations in the deterioration of the peritoneum during dialysis. The imbalance between these situations may cause fibroproliferative diseases and could be an important cause of morbidity and mortality.

In this review we discuss the implication of Th17 and Treg cells in kidney function and fibrotic diseases in different animal experimentation models. More specifically we will focus on the origin of peritoneal damage and its relationship with the intraperitoneal presence of these particular subsets of T lymphocytes, as well as on its clinical implication in peritoneal damage in PD patients, which frequently results in an inability to continue with the treatment.

## 2. T Helper 17 and Regulatory T Cell Differentiation and Their Plasticity

Th17 cells represent a subset of T helper cells that secrete mainly interleukin- (IL-) 17 as well as other proinflammatory cytokines, and they have been related to many autoimmune and chronic inflammatory diseases [29]. There is a balance between Th17 and Treg cells that depends on the activation of the transcription factor ROR*γ*t (factors retinoic acid receptor-related orphan receptor *γ*t) and Stat3 (signal transducer and activator of transcription 3), or FoxP3 (forkhead box P3) and Stat5, respectively, which regulate the immune response through the secretion of pro- and anti-inflammatory cytokines [30–33]. On the other hand, the importance of Treg cells to the maintenance of peripheral tolerance under noninflammatory conditions throughout life has also been confirmed. In fact, mice lacking Treg cells presented a fatal inflammatory response [34, 35].

**Figure 2:**
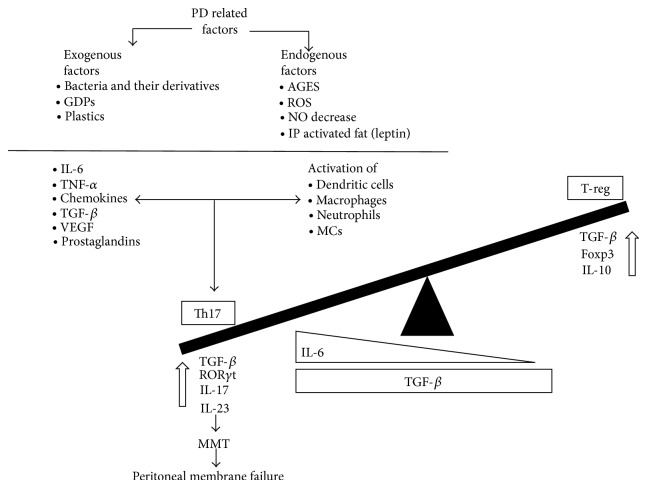
Mechanisms of Th17/Treg-balance in the peritoneal cavity in PD. Th17 predominance. In the peritoneal cavity local factors activate dendritic cells, macrophages, and neutrophils to produce proinflammatory molecules (IL-6, TNF*α*, TGF-*β*, VEGF, chemokines, and prostaglandins). Immune cells and proinflammatory molecules are reciprocally activated; thus cells induce proinflammatory molecule release and vice versa. These molecules activate Th17 cells and IL-17 production, which may result in fibrosis and MMT. This occurs in the presence of continuously high TGF-*β* levels. In contrast, in the presence of inactive immune cells together with low IL-6 levels, Treg cells are activated, releasing IL-10 and TGF-*β* that may block MMT. Abbreviations: GDPs: glucose-derived products. IP: intraperitoneal. AGEs: advanced glycation end products.

The main cytokines involved in Th17/Treg balance are the TGF-*β* (transforming growth factor beta) and IL-6 (interleukin-6) [27, 36, 37]. IL-6 is strongly induced in cells of the innate immune system upon stimulation of pattern recognition receptors such as toll like receptors (TLR) or C-type receptors. It has been shown that mice lacking IL-6 present a deficiency in the differentiation of effector T cells [38, 39]. TGF-*β* in the absence of IL-6 induces Foxp3, thus pushing T-cell differentiation away from the Th17 transcriptional program and decidedly toward the Treg lineage [33]. Moreover, in the central nervous system, TGF-*β* without the synergy of IL-6 will force T cells to differentiate through the T regulatory cell lineage [40]. In contrast, the proinflammatory cytokine IL-6 in the absence of TGF-*β* activates Stat3 by phosphorylating it, which overcomes Foxp3 inhibition of ROR*γ*t transcriptional activity. This process leads to the upregulation of the IL-23R, thus pushing T-cell differentiation toward a Th17 fate [33]. Therefore the cytokine environment is essential for the predominance of an inflammatory or an anti-inflammatory response (Figure 2).

## 3. Th17 and Tissue Fibrosis

Fibroproliferative diseases such as idiopathic pulmonary, liver, cardiovascular, and renal fibrosis are usually associated with chronic inflammation, as has been described previously [41–45]. When an inflammatory response becomes chronic, the accumulation of ECM is more extensive and the function of the organ is compromised. A number of studies have highlighted the roles of Th17/Treg/Th1/Th2 responses in the pathogenesis of tissue fibrosis [46, 47]. Among these responses, Th17 cells may mediate strong inflammation by producing a cocktail of cytokines such as IL-6, IL-17A, IL-17F, and IL-22, among which IL-17A has been characterized as the major effector cytokine in causing a sustained inflammatory response.

Recent studies have investigated the role of Th17 response in fibrosis. It has been reported that administration of IL-17A* in vitro* increased the synthesis and secretion of collagen in alveolar epithelial cells in a pulmonary fibrosis model. Moreover, all IL-17-associated signaling pathways were mainly activated in fibrotic lung biopsies, and a blockade of IL-17A attenuated tissue injury, inflammation, and fibrosis in acute and chronic injuries [48].

Furthermore, IL-17 has also been reported to be involved in the pathogenesis of chronic liver fibrosis [44, 49–51]. The same mechanism was proposed in another study on liver damage in chronic hepatitis B patients who presented an elevated Th17 cells population [52].

Th17 cells also play a crucial role in autoimmune myocarditis, as based on* in vitro* and* in vivo* experiments which confirm that IL-17 induced cardiac fibrosis by activating the protein kinase C-*β*/Erk1/2/NF-*κ*B pathway [53]. Moreover, the regulatory molecule CD69, through the regulation of Th17 effector responses, limits myocardial inflammation, fibrosis, and subsequent heart failure [54].

Recent studies have shown the importance of Th17 cells, and the hallmark cytokine IL-17A, in immune-mediated glomerulonephritis, including experimental antimyeloperoxidase glomerulonephritis, crescentic glomerulonephritis, and lupus nephritis [55, 56]. Th17 cells participate in renal damage, as demonstrated by an experimental study in mice showing that Th17 cell injection caused albuminuria and neutrophils infiltration in the kidney [57]. Recent studies also show the presence of Th17 cells and elevated renal production of IL-17A in nonimmune experimental renal diseases, including a model of unilateral ureteral obstruction [58]. In experimental ischaemia reperfusion, neutrophils, but not Th17 cells, were the main sources of IL-17A and contribute to renal injury by natural killer T activation and IL-12/IFN-*γ* production [59]. In renal allograft rejection, positive staining for IL-17A has been detected in tubular cells [60], as we have observed in an experimental model of CCN2-mediated renal damage, suggesting that renal cells could produce this cytokine and contribute to extending the damage. With this model we recently demonstrated that a blockade of IL-17A diminished renal inflammation [58].

Based on all the above studies, IL-17 has been proposed as a drug target in many fibrotic diseases [61].

## 4. Regulatory T Cells and Tissue Fibrosis

Although an induced Th17 response is connected to fibrogenesis, a relative decrease in the number of Treg cells may also be involved in the pathogenesis of inflammatory and fibrotic diseases. The evaluation of these cells in the context of experimentally induced fibrosis has been challenging, and Treg depletion in mice has been demonstrated to attenuate the development of lung fibrosis [62]. Moreover, in an experimental animal model of cardiac fibrosis, the depletion of Treg cells and/or adoptive transfer of isolated Tregs ameliorated cardiac fibrosis, indicating a protective role of regulatory T cell in tissue fibrosis [63]. Recently, it has been reported that Treg cells are essential for preventing from accumulation of fibrocytes and collagen deposition in a pulmonary disease animal model. In this study, it was shown that a blockade of Treg cells increased the accumulation of solid collagen and progression of the disease [64]. Finally, in another study (Keloid fibrotic disease) the potential role of Treg cells in attenuating collagen synthesis was investigated. This group found that the imbalance of Tregs may contribute to the development of this fibrotic disease and that the correction of this imbalance may be of therapeutic value [65].

## 5. Th17 and Treg Lymphocyte Subsets in the Peritoneal Cavity on PD

PD-related factors locally stimulate Th17 cells and can be subdivided into two groups: exogenous and endogenous. The exogenous factors include bacteria and their derivatives, which enter into the peritoneal cavity through PD-catheter or via intestinal translocation and can provoke peritonitis episodes [66]. Peritoneal endogenous factors such as AGEs [67] could be involved in the induction of IL-17 levels by activating IL-6 and TGF-*β* proinflammatory cytokines, respectively. Although there is little data regarding how advanced glycation end products (AGEs) are implicated in peritoneal dialysis damage, their implication in posttransplantation and diabetic kidneys showed an induction of IL-6 and TGF-*β*, which are promoters of Th17 differentiation (Figure 2). Thus, it is plausible that the induced Th17 activity may have a poor fate in peritoneal damage during peritoneal dialysis. In nondiabetic PD patients, an elevation of IL-17 in peritoneal cavity effluents followed by a peritonitis episode was demonstrated [66], which is one of the main complications that lead to peritoneal fibrosis in PD patients [3].

Currently, it is accepted that mesothelial to mesenchymal transition (MMT) is a key process in PM survival in PD. Mesothelial cells lose their basolateral and basoapical polarity, acquiring a fibroblastoid phenotype with migration capacity. The cells invade the submesothelial compact zone where they synthesize ECM and VEGF, which are responsible for fibrosis and angiogenesis, respectively [68, 69]. IL-17 itself is capable of inducing epithelial to mesenchymal transition in bronchial cells [70]. Although this effect has not been demonstrated in the peritoneum, it is very likely that IL-17 also contributes to PM deterioration via MMT induction.

The definitive evidence on IL-17 involvement in PM damage on PD was recently provided by Rodrigues-Díez et al. (2014). This study demonstrated in both mice and human samples that IL-17 is overexpressed in peritoneal biopsies. This was the first report to demonstrate that IL-17 participates in the typical fibrotic changes suffered in PM during long-term PD (induced fibronectin, *α*-smooth muscle actin, and fibroblast specific protein-1 expression). Moreover, to better elucidate the effects of IL-17 on PM, intraperitoneal IL-17 was injected in mice, reproducing the changes that normally take place in PD patients. On the other hand, the use of a neutralizing IL-17A antibody injected intraperitoneally in mice exposed to PDF for 35 days blockaded the anatomical changes in the PM and reduced peritoneal fibrosis [71].

On the other hand, Treg cells are strongly connected with immune tolerance. Patients in end-stage renal disease with their suppressed immune response suffer impaired Treg cell responses [72]. Currently, there is not much evidence regarding the role of Treg cells in the peritoneal cavity during PD. To our knowledge, there is one study focusing on the function of Treg cells on peritoneal damage. This study concluded that rosiglitazone, a PPAR*γ* agonist, augments the intraperitoneal IL-10 levels (Treg-associated cytokine), increases the recruitment of CD4^+^ CD25^+^ FoxP3^+^ (regulatory T cells), and finally attenuates peritoneal fibrosis in an experimental mouse PD model [73].

Evidence suggests that Th17 cells share common progenitors with Treg cells and that the developmental pathways of these two subsets are reciprocally regulated [74]. In fact, it has been recently demonstrated that a reduction in IL-17 secretion due to Treg cell activation is associated with a diminished fibrotic response specifically in PD. The vitamin D pathway has been shown to regulate inflammatory responses. In regard to this, the effect of paricalcitol, a vitamin D receptor activator, was tested in a mouse model of PD to evaluate its effect on inflammatory cells and on the outcome of peritoneal fibrosis. It was found that the group that was treated with PDF presented increased levels of IL-17 cytokine in the peritoneal effluents compared with the group that was treated with paricalcitol diluted in the PDF. Moreover, the increased IL-17 concentration was perfectly correlated with the thickness of the peritoneum, meaning IL-17 is a profibrotic cytokine. This effect was related to an increased number of Tregs in the group that was treated with paricalcitol [75].

## 6. Therapeutic Approaches to Prevent Peritoneal Damage Using the Th17/Treg-Axis: From Animal Models to PD Patients

Accepting the evidence above that Th17 and Treg subsets are involved in peritoneal damage in PD, we propose the following therapeutic strategies.

### 6.1. Use Drugs and Molecular Strategies

#### 6.1.1. mTOR Inhibitors

**Figure 3:**
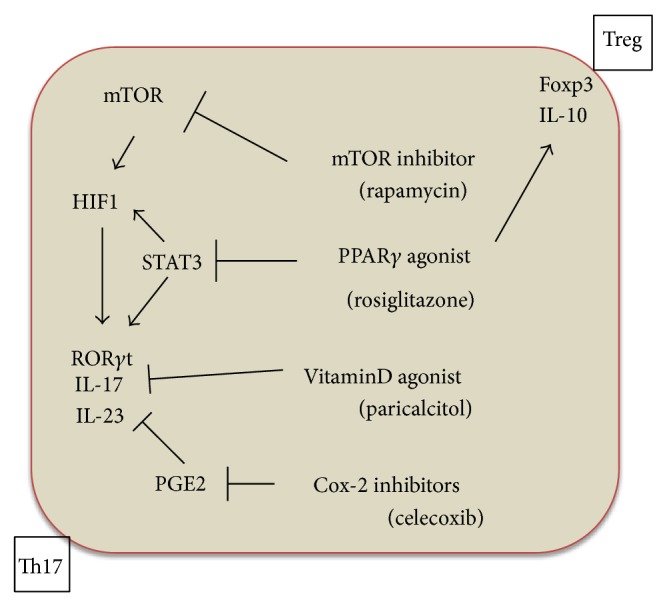
Therapeutic approaches to prevent peritoneal damage using the Th17/Treg-axis as a target. One of the most important modulators of Th17/Treg activity is the concentration of branch chain amino acids (BCAA). Although the data in relation to BCAA plasma levels in a uraemic state are contradictory, many articles indicate that these amino acids are decreased in uraemia due to systemic acidosis, inflammation, amino acid misbalances, and liquid overload. Normal or relative normal BCAA levels activate an mTOR (mammalian Target of Rapamycin) cascade including HIF1 and ROR*γ*t and subsequently IL-17 and IL-23 production. Rapamycin, an mTOR inhibitor, would block this cascade, thus providing an anti-inflammatory/antifibrotic and possibly anti-MMT effect. Moreover, PPAR*γ* agonists can also inhibit Th17 differentiation through a direct blockade of Stat3 transcription factor and HIF-1. Ultimately ROR*γ*t is downregulated, and IL-17 and IL-23 production is decreased. However the PPRA*γ* agonists are also able to act on the anti-inflammatory cascade. In the peritoneal cavity in PD, rosiglitazone augments IL-10 levels and Treg activity (upregulation of FoxP3^+^). This process could be one of the most important mechanisms by which PPRA*γ* agonists protect the PM. In addition, paricalcitol, a specific vitamin D activator, has been recently shown to inhibit IL-17 production and PM fibrosis and possibly decrease MMT. Finally, celecoxib, a Cox2 inhibitor, decreases IL-17 production by blocking the E2 prostaglandin levels and thus attenuates the PM damage induced by PDF.

The mTOR inhibition by Rapamycin may diminish IL-17 production. The mTOR activation induces hypoxia induced factor-1 (HIF-1) and ROR*γ*t activation and subsequently IL-17 and IL-23 production [76]. Thus, these drugs may provide an anti-inflammatory/antifibrotic effect and possibly an anti-MMT action as was demonstrated by Aguilera et al. [77] (Figure 3).

#### 6.1.2. Peroxisome Proliferator-Activated Receptors- (PPAR-) *γ* Agonists

The use of PPAR*γ* agonists, for example, rosiglitazone, may be a therapeutic alternative to prevent peritoneal damage [78]. These receptors show a double protective effect. First, they inhibit the Th17 differentiation via a Stat3 cascade blockade, which results in a downregulation of ROR*γ*t and a decrease in IL-17 production [79]. Moreover, in an animal model study in which the effect of rosiglitazone in the preservation of the peritoneal membrane was investigated, it was found that rosiglitazone augmented the intraperitoneal IL-10 levels (Treg-associated cytokine) and increased the recruitment of CD4^+^ CD25^+^ FoxP3^+^ (regulatory T cells) [73] (Figure 3).

#### 6.1.3. Vitamin D Receptor Activators

Another interesting drug that could ultimately have a beneficial effect on PM in PD is paricalcitol. Using a mice PD model, it was demonstrated that the PM thickening was reduced in mice treated with paricalcitol in comparison with the nontreated group. Moreover, in the effluents of mice treated with paricalcitol, an increased number of Treg cells and lower IL-17 levels were found in comparison with the nontreated group. In addition, the IL-17 levels measured in peritoneal effluent showed a positive linear correlation with the PM thickness [75]. These data suggest a direct involvement of IL-17 in peritoneal injury and paricalcitol may act on this target (Figure 3).

#### 6.1.4. COX-2 Inhibitors

Celecoxib, a cyclooxygenase- (Cox-) 2 inhibitor agent, was shown to prevent the PM damage in PD when administrated orally to a group of mice in PD, acting directly on the inflammatory cascade in general. After 5 weeks of treatment, the celecoxib group showed a fibrotic response similar to healthy controls, while the untreated group exposed to PD only developed considerable fibrosis [19] (Figure 3).

## 7. Discussion

One of the most devastating complications of PD treatment is peritoneal fibrosis. The endogenous and exogenous factors mentioned above related to PDF generate a chronic inflammatory response in the peritoneal cavity. The balance between Th17 and Treg cells is guided by proinflammatory cytokines secreted from Th17 cells and anti-inflammatory cytokines produced from regulatory T cells. Any factor that may alter this balance can lead to peritoneal deterioration and finally to peritoneal damage. The high levels of various proinflammatory molecules such as IL-6 and TGF-*β* cytokines during PD create an environment that induces a chronic inflammatory condition in the peritoneal cavity and generates peritoneal fibrosis. This emergent concept suggests that the immune imbalance is the fundamental key for PM deterioration in PD.

Moreover, Th17 signaling with high IL-17 levels has been implicated in the aetiology of several types of inflammatory and fibrotic diseases. Therefore, components of the IL-17 and Treg cells pathway are considered highly “druggable” and are important targets for the treatment of these inflammatory and fibrotic diseases. Current evidence indicates that IL-17 inhibition and Treg activation are logical therapeutic strategies for the treatment of animal peritoneal fibrosis [71, 73, 75].

In conclusion, the importance of developing new therapies to protect the peritoneal membrane blocking the IL-17 secretion or activating the Treg pathway has been demonstrated. Some novel therapeutic strategies tested in animal models and* in vitro* include the administration of m-Tor inhibitors, PPAR*γ* agonists, vitamin D receptor activators, and Cox-2 inhibitors. Lessons learned from regulators of Th17/Treg-axis may aid in the future clinical implementation of these agents with the goal of reducing peritoneal fibrosis and improving patients' life undergoing PD.

## 8. Conclusion

PD-related factors are responsible for Th17 activation/Treg deactivation in the peritoneal cavity during peritoneal dialysis. The Th17/Treg-axis is important for maintaining the anatomical and functional integrity of the PM. Therefore, the Th17/Treg-axis may be considered a future therapeutic target.
