# Corrigendum to “T Helper 17/Regulatory T Cell Balance and Experimental Models of Peritoneal Dialysis-Induced Damage”

**DOI:** 10.1155/2017/6130208

**Published:** 2017-02-08

**Authors:** Georgios Liappas, Guadalupe Tirma Gónzalez-Mateo, Pedro Majano, José Antonio Sánchez-Tomero, Marta Ruiz-Ortega, Raquel Rodrigues Díez, Pilar Martín, Raquel Sanchez-Díaz, Rafael Selgas, Manuel López-Cabrera, Abelardo Aguilera Peralta

**Affiliations:** ^1^Department of Immunology, Centro de Biología Molecular Severo Ochoa (CBM), Consejo Superior de Investigaciones Científicas (CSIC), 28049 Madrid, Spain; ^2^Department of Nephrology, Instituto de Investigación del Hospital Universitario La Paz (IdiPAZ), 28046 Madrid, Spain; ^3^Department of Molecular Biology, Instituto de La Princesa (IP), 28006 Madrid, Spain; ^4^Nephrology Service, Hospital Universitario de La Princesa (IP), 28006 Madrid, Spain; ^5^Laboratory of Cellular Biology and Rare Diseases, Fundación Jiménez Díaz, Universidad Autónoma de Madrid (UAM), 28040 Madrid, Spain; ^6^Department of Molecular and Vascular Inflammation, Centro Nacional de Investigaciones Cardiovasculares (CNIC), 28029 Madrid, Spain

In the article titled “T Helper 17/Regulatory T Cell Balance and Experimental Models of Peritoneal Dialysis-Induced Damage” [1], there was an error in the Acknowledgments section, which should be corrected as follows.

This work was supported in part by grants from Ministerio de Economia y Competitividad SAF2010-21249 to Manuel López-Cabrera, Comunidad Autónoma de Madrid 2010-BMD2321 (FIBROTEAM) to Manuel López-Cabrera, and Fondo de Investigaciones Sanitarias RETICS 06/0016 and PI 09/0064 to Rafael Selgas and from Fondo de Investigaciones Sanitarias (FIS)-FEDER funds, PI 12/01175 Instituto Carlos-III to Abelardo Aguilera Peralta. Georgios Liappas is fully supported from European Union, Seventh Framework Program “EuTRiPD,” under Grant Agreement PITN-GA-2011-287813. The authors would like to thank Juliette Siegfried and her team at ServingMed.com for editing the language of the paper.
